# Resilience and its associated factors in head and neck cancer patients in Pakistan: an analytical cross-sectional study

**DOI:** 10.1186/s12885-021-08624-8

**Published:** 2021-08-03

**Authors:** Nida Zahid, Wajeeha Zahid, Wardah Khalid, Iqbal Azam, Mubasher Ikram, Aneesa Hassan, Haissan Iftikar, Shireen Shehzad Bhamani, Adnan Abdul Jabbar, Shabbir Akhtar, Moghira Iqbaluddin Siddiqui, Mohammad Sohail Awan, Nargis Asad, Khabir Ahmad

**Affiliations:** 1 Department of Surgery, Aga Khan University Hospital, Stadium Road, Karachi, 74800 Pakistan; 2 Department of Community Health Sciences, Aga Khan University Hospital, Stadium Road, Karachi, 74800 Pakistan; 3 School of Nursing and Midwifery, Aga Khan University Hospital, Stadium Road, Karachi, 74800 Pakistan; 4 Department of Oncology, Aga Khan University Hospital, Stadium Road, Karachi, 74800 Pakistan; 5 Department of Psychiatry, Aga Khan University Hospital, Stadium Road, Karachi, 74800 Pakistan

**Keywords:** Anxiety, Developing countries, Depression, Head and neck cancer, mental health, Psycho-oncology, Resilience, Social support, Oncology

## Abstract

**Introduction:**

The study aimed to assess resilience and its associated factors in head and neck cancer patients, post-treatment in a low middle income country (LMIC) such as Pakistan.

**Methods:**

An analytical cross-sectional study was conducted from November 2019 to May 2020 among head and neck cancer patients aged at least 18 years at the largest private tertiary care hospital, in Karachi, Pakistan. Information regarding their resilience scores was collected through Wagnild and Young’s Resilience scale that comprises of 14 items (RS-14). Moreover, depression and anxiety were also assessed via Hospital Anxiety and Depression Scale (HADS) and social support was assessed by Enriched Social Support Instrument (ESSI).

**Results:**

The data was analyzed by linear regression modeling. Unadjusted and adjusted beta coefficients with 95% CI were reported. A total of 250 head and neck cancer patients were recruited, 79% of them were males. Mean age of the patients was 51.59 years with 93% having high social support and only 8% having severe depression and 3% having severe anxiety. After adjusting for the covariates in multivariable analysis resilience was associated with severe depression (− 17[− 20.98,-12.93]) or borderline depression (− 4[− 8.41,-0.39]), severe anxiety (− 11 [− 17.88,-4.18]), low social support (− 6[− 9.62,-1.71]), having family members of > 6 in the household (− 2[− 4.31,-0.29), smokeless tobacco users post- treatment (10[5.79, 14.45]), and those who underwent tracheotomy (− 4[− 7.67,-0.21]). There was a significant interaction between education and role in the family (decision maker).

**Conclusion:**

In Pakistan, a South Asian LMIC, collectivist culture prevails, family ties are greatly promoted thus resilience and social support is highly prevalent in head and neck cancer patients resulting in lower prevalence of depression and anxiety. Our study highlights that higher resilience is prevalent among small families less than six members, as the welfare of the individual is prioritized over multiple needs of the family. Formal Education and role in household/decision making power are effect modifiers in our study, demonstrating its protective effect on the mental health of head and neck cancer patients. High resilience scores were reported among current smokeless tobacco users as compared to quitters post treatment. Resilience-building interventions should be formulated to aid head and neck cancer patients to cope with the disease and its sequel.

**Supplementary Information:**

The online version contains supplementary material available at 10.1186/s12885-021-08624-8.

## Introduction

Cancer is the leading cause of mortality in the developed world and second most common in developing countries [1]. Head and neck cancers ranks sixth with approximately 630,000 diagnosis, causing 350,000 deaths annually [2] with cancer of oral cavity being the highest contributor to its prevalence [3]. The risk of developing head and neck cancer increases 10 fold among habitual tobacco users than non-users [4]. The highly prevalent consumption of smokeless tobacco in our part of the world further adds to these numbers ranking it as the second most common cancer in Pakistan [5, 6]. The frequent consumption of pan, gutka, supari which contains highly carcinogenic products such as betel quid, areca nut (with or without tobacco), slaked lime, pre-disposes our population to oral cancer [7] with approximately 18,880 new cases annually. Lip and oral cavity cancer account for majority of the head and neck cancers in Pakistan having higher incidence in males (15.9% new cases) in comparison to females [5].

Head and neck cancer surgeries are mutilating, leaving both physical and functional defects which have a huge bearing on a patients’ psychosocial well-being resulting in anxiety and depression [8] which in turn leads to low resilience [9, 10]. Resilience is the ability of an individual to positively adapt and maintain or regain emotional stability while experiencing an adverse condition [11]. Resilience might depend on several factors including positive emotions, cognitive flexibility (such as acceptance), active coping style, and spirituality etc. Resilience has shown to play a protective role against distress in cancer patients [12–16]. If improved psychological well-being is considered an endpoint of medical care along with survival, mortality, morbidity then resilience could be considered as a fulcrum between cancer symptoms and patient distress [17]. According to recent data, the prevalence of head and neck cancer is escalating in Pakistan, and limited information is available regarding resilience of head and neck cancer in our setting, a low middle-income country (LMIC) having diverse cultural, social and economic factors [18]. This study will bridge this gap and its findings would be beneficial in formulating targeted interventions as per need to improve resilience.

The aim of the study is to determine resilience in head and neck cancer patients at a tertiary care hospital in Pakistan, and to investigate its relationship with patients’ socio-demographic factors, clinical characteristics, social support, and mental health.

## Methods

### Study design

An analytical cross-sectional study was conducted from November 2019 to May 2020 among head and neck cancer patients aged at least 18 years at Aga Khan University Hospital (AKUH), the largest private tertiary care hospital, in Karachi, Pakistan. At one point in time, data on resilience (outcome variable) and other predictor variables were obtained from head and neck cancer patients. The information was gathered on a structured questionnaire on the patients’ socio-demographic characteristics, and validated instruments were used to evaluate the head and neck cancer patients’ resilience, depression, anxiety, and social support status.

### Study participants

Head and neck cancer patients, 18 years and above, currently ≥4 weeks post-initiation of treatment at AKUH, living in Pakistan since the past 3 months and who provided written informed consent were included in the study. We excluded patients with physical comorbidities, (stroke, renal failure), as these debilitating diseases would distort the study results. Patients suffering from any known psychotic disorders (eg, manic disorder, schizophrenia etc), that lead to cognitive inability or require medication (such as antidepressants) were excluded. Patients with co-morbidities such as Cardiovascular Diseases (CVD), Diabetes Mellitus or Chronic Obstructive Pulmonary Disease (COPD) weren’t excluded as their prevalence in Pakistani population is very high- [19] and their ineligibility would hinder in achieving the required sample size, therefore these confounders were adjusted during analysis [20].

### Sample size and sampling strategy

Nonprobability purposive sampling technique was employed for selecting the patients. During the study period of November 2019 to May 2020, trained research assistants approached all head and neck cancer patients visiting the surgical/oncology clinics at AKUH as per their scheduled appointments. Potential participants were screened for eligibility by trained research assistants. The eligible participants were briefed on the scope and nature of the study, as well as the extent of their participation. Patients who provided written informed consent for participation were enrolled in the study and the study questionnaire was administered to them by the research assistants. The questionnaire was administered to each patient for about 30 to 40 min.

The minimum sample size was calculated to be 250 based on mean resilience scores for head and neck cancer patients from previous studies [21–25]. It was calculated using one population mean formula, based on a SD range of 16.5–40.8, 5% level of significance with precision of 2.5, and adding non-response of 10%.

### Data collection

Prior to participant recruitment, the questionnaire was pretested on 5% of the sample size to identify any ambiguities. The final questionnaire was administered by research assistants in Urdu (Pakistan’s national and official language), and it was divided into three sections:

### Outcome variable

#### Resilience

Data regarding resilience was collected through the validated Urdu version of the Wagnild and Young’s Resilience scale comprising of 14 items (RS-14), and each item was assigned a 7-point likert scale ranging from 1 (strongly disagree) to 7 (strongly agree). The RS-14 assessed five core characteristics of resilience; purposeful life, perseverance, equanimity, self-reliance and existential loneliness [26]. Its test–retest correlation coefficient was 0.49 and Cronbach’s alpha was 0.76. The concurrent validity was 0.813 [27].

### Predictors

#### Socio-demographic and clinical characteristics

The questionnaire addressed patients’ age, gender, ethnicity, education, family status, comorbidities conditions (hypertension, diabetes, cardiovascular disease), history of addictions (including smoking, substance abuse), employment status of patient and family members and monthly household income. Data on major recent life altering events was collected. Data regarding clinical characteristics; type of tumor, surgery, chemotherapy and/or radiotherapy and site of tumor was also collected.

#### Psychosocial characteristics

Data regarding depression and anxiety was collected via the validated Urdu version of Hospital Anxiety and Depression Scale (HADS) which comprises of 14 items, equally subdivided into the anxiety and depression subscales with each item scored from 0 to 3 [28, 29]. An individual who scored 8 to 10 was classified as mildly anxious and depressed whereas the one scoring at least 11 was classified as anxious and depressed.

Data regarding social support (functional and emotional) was collected through the validated Urdu version of Enriched Social Support Instrument (ESSI) [9] with a CVI for relevance of 0.95, clarity of 0.97, and Cronbach’s alpha of 0.82 [30]. It comprises of 7 items and an aggregate score of at most 18 was considered as low social support.

### Plan of analysis

The data was analyzed on STATA version 15. Continuous variables were reported as mean ± standard error (SE)/median (IQR), while categorical variables were reported as frequency and percentages. Linear regression was used to report unadjusted and adjusted beta coefficients with 95% CI, to determine the factors associated with resilience among head and neck patients. The dependent variable was resilience. The independent variables were demographic variables (age, gender, monthly income, working status), comorbidities (hypertension, diabetes Mellitus, CVD), addictive substance use (tobacco and alcohol use), family history of cancer, tumor- and treatment-related factors (type of tumor, type of surgical intervention, and adjuvant therapy), social support, depression and anxiety. The plausible interaction that we assessed was formal schooling and role in the family. A *p*-value < 0.05 was considered significant for all analyses.

### Ethical considerations

The study was approved by the Ethical Review Committee of the Aga Khan University Hospital. Written informed consent was obtained from all the study participants. All the patients’ information was kept confidential and no personal identifier was disclosed. Participants identified as depressed via the Hospital Anxiety and Depression Scale (HADS) were provided on-the-spot counseling by a trained psychologist. Whereas, those identified with severe depression (having suicidal thoughts) were referred to seek advice of a psychiatrist.

## Results

### Description of the study participants

The Table 1 describes the socio-demographic factors, co-morbidities, addictive use, family history, tumor related factors, social support and mental health of the head and neck cancer survivors. The mean age of the participants was 51.59 (0.83) with 79% males. 87% of the participants had acquired formal education and mean years of schooling was 11.25(0.23) years. The mother tongue of about 50% of the participants was Urdu. Majority (87%) of the participants were married and 53% of them lived in extended families. About 52% of the participants had ≤6 household family members while 48% had more than 6 family members. 55% of the participants were head / decision makers of the family. More than half (64%) of the participants were not currently working and about 73% participants spouse were not employed. The median household monthly income was PKR 45000 (22650–100,000) and 20% of the participants had their own business.

**Table 1:** Description of Head and Neck Cancer Survivors (*n* = 250)

Participants’ Sociodemographic Characteristics	
Variables	***N*** = 250 N (%)/ Mean ± SE
**Age (years)**	51.59 ± 0.83
**Gender**	
**Male**	198 (79.2)
**Female**	52 (20.8)
**Formal Schooling**	
**Yes**	218 (87.2)
**No**	32 (12.8)
**Informal Schooling**	
**Yes**	69 (27.6)
**No**	181 (72.4)
**Type of Informal Schooling**	***N*** **= 69**
**Madrassa**	42(60.9)
**Self-Learnt (reading)**	2 (2.9)
**Learnt Quran**	21 (30.4)
**Multiple**	4 (5.8)
**Mother Tongue**	
**Urdu**	117 (46.8)
**Sindhi**	46 (18.4)
**Punjabi**	14 (5.6)
**Pushto**	10 (4.0)
**Memoni**	16 (6.4)
**Baloch**	12 (4.6)
**Kachi**	7 (2.8)
**Other**	21 (8.4)
**Marital Status**	
**Married**	218 (87.2)
**Single**	14 (5.6)
**Other**	18 (7.2)
**Family Structure**	
**Extended**	134 (53.6)
**Nuclear**	116 (46.4)
**Role in Family**	
**Head/Chief Decision-Maker**	138 (55.2)
**Not Head; involved in decisions**	98 (39.2)
**Not Head; not involved in decisions**	14 (5.6)
**Number of Members in Family**	
**≤ 6**	129 (51.6)
**> 6**	121 (48.4)
**Working Status**	
**Working**	89 (35.6)
**Not Working**	161 (64.4)
**Spouse’s Working Status**	***N*** **= 218**
**Working**	36(16.5)
**Not Working**	182 (83.5)
**Monthly Household Income (PKR/USD)**	
**No Income**	18 (7.2)
**2000–25,000 ($ 13-$151)**	40 (16.0)
**25,000–40,000 ($151–$242)**	26 (10.4)
**40,000–80,000 ($242–$484)**	69 (27.6)
**80,000–170,000 ($484–$1028)**	97 (38.8)
**Household Income (PKR/USD)**	45,000 (22650–100,000)/ 281(142–625)
**Comorbidities**	
**Hypertension (HTN)**	64 (25.6)
**Diabetes (DM)**	39 (15.6)
**Cardiovascular Disease (CVD)**	10 (4.00)
**Undergone any disease**	63 (25.2)
**Addictive use Smoked tobacco users**	
**Current**	5 (2.4)
**Ex**	59 (23.6)
**No**	185 (74.0)
**Smokeless tobacco users**	
**Current**	14 (5.6)
**Ex**	103 (41.2)
**No**	133 (53.2)
**Family History of Cancer**	
**Family history of head and neck cancer**	32 (12.8)
**Family History of any cancer other than head and neck cancer**	38 (15.2)
**Family history of depression**	10 (4.0)

*PKR* Pakistani Rupee

*USD* US Dollars

We observed that 26% had hypertension, 15% had Diabetes and 4% had CVD. About 2.4% of the participants were current smokers and 6% were current smokeless tobacco users, however 24% were ex-smokers and 41% were ex smokeless tobacco users. 13% of the participants had a family history of head and neck cancer while 15% had family history of other type of cancers. Only 4% of the participants had a family history of depression. 7% of the participants reported death in their family in the last 6 months.

Table 2 indicates the tumor and treatment related factors. Majority of the participants (82%) had oral cancer. 41% had feeding tube while 8% had tracheotomy. Majority of the participants (53%) received combination adjuvant therapy (chemotherapy and radiotherapy).

**Table 2:** Tumor- and Treatment-related Factors

Variables	***N*** = 250 N (%)
**Tumor Type**	
**Oral Cancer**	205 (82)
**Laryngeal**	35 (21.2)
**Others**	10 (4.0)
**Surgical Intervention**	
**Only Biopsy**	187 (74.8)
**Only Total Resection**	7 (2.8)
**Multiple Interventions**	4 (1.6)
**No surgical intervention**	52 (20.8)
**Adjuvant Therapy**	
**Chemotherapy**	12 (4.8)
**Radiotherapy**	46 (18.4)
**Combination**	132 (52.8)
**No Adjuvant Therapy**	60 (24.0)
**Treatment Stage for head and neck cancer**	
**On-going**	108 (43.2)
**Complete**	142 (56.8)
**Feeding Tube Needed**	104 (41.6)
**Tracheostomy Needed**	19 (7.6)

Table 3 shows the social support, mental health and resilience among the head and neck cancer patients. We observed that 7% of the participants had low social support. Moreover, 6% reported borderline depression and 8% had severe depression. 3% of the participants had borderline anxiety and 3% had severe anxiety. The mean resilience score was 82 ± 0.65.

**Table 3:** Social Support, Mental Health and Resilience

Variables	***N*** = 250 N (%)/Mean ± SE
**Social Support**	
**≤ 18 (Low Social Support)**	18(7.2)
**> 18 (High Social Support)**	232 (92.8)
**Depression**	
**0–7 (Normal)**	215 (86.0)
**8–10 (Mild Depression)**	16 (6.4)
**11–21 (Symptomatic Depression)**	19 (7.6)
**Anxiety**	
**0–7 (Normal)**	237 (94.8)
**8–10 (Mild Anxiety)**	7 (2.8)
**11–21 (Symptomatic Anxiety)**	6 (2.4)
**Resilience**	8 (3.2)
**< 65 (Low Resilience)**	79 (31.6)
**65–81 (Moderate Resilience)** **> 81 (High Resilience)**	163 (65.2)
**Resilience**	81.57 ± 0.65

### Univariate and multivariable analysis to assess resilience and its associated factors among head and neck cancer survivors

The Table 4 shows the univariate and multivariable analysis to assess resilience and its associated factors among head and neck cancer survivors.

**Table 4:** Univariate and Multivariable Analysis to Assess Resilience and Its Associated Factors among Head and Neck Cancer Survivors (*n* = 250)

		4a.Univariate Analysis		4b. Multivariate Analysis		
Variables		Unadjusted Beta Coefficient (SE)	95% Confidence Interval (CI)	Adjusted Beta Coefficient (SE)	95% Confidence Interval (CI)	
**Formal Schooling**	Yes (ref)					
	No	- 3.37(1.93)	(−7.18,0.42) *	−0.66(2.04)	(− 4.67,3.36)	
**Years of schooling**		0.24(0.18)	(−0.11,0.60) *	NS	NS	
**Role in family**	Head/ decision makers of the family (ref)	–	–	–	–	
	Not Head /decision makers of the family	2.41(1.34)	(−0.288,5.05)*	1.86(1.11)	(− 0.33,4.05)	
	Not head/ not the decision makers of the family	−4.59(2.84)	(−10.21,1.01)*	0.67 (2.41)	(−4.07, 5.42)	
**Family members in the household**	Less than 6 (ref)	–	–	–	–	
	More than 6	−0.18 (1.30)	(−2.74,2.38)*	−2.30 (1.02)	(−4.31,-0.29)**	
**Cardiovascular Disease**	Yes	4.3 (3.30)	(−2.20, 10.80)*	NS	NS	
	No (ref)					
**Smokeless Tobacco user**	Yes	9.71(2.86)	(4.06,15.35)*	10.12(2.20)	(5.79,14.45)**	
	No	0.855 (1.32)	(−1.74,3.45)	1.06 (1.03)	(−0.97, 3.08)	
	Ex user (ref)	–		–		
**Family History of any cancer**	Yes	- 4.15(1.79)	(−7.67,-0.62)*	NS	NS	
	No (ref)					
**Family history of depression**	Yes	- 5.49(3.29)	(−11.98,1.00)*	NS	NS	
	No (ref)					
**Deaths in the family**	Yes	3.81(2.50)	(−1.11,8.74)*	NS	NS	
	No (ref)					
**Patient has Feeding tube**	Yes	- 2.90 (1.30)	(−5.47, −0.33)*	NS	NS	
	No (ref)					
**Patient has tracheotomy**	Yes	- 6.99 (2.41)	(−11.74, −2.24)*	- 3.98 (1.92)	(−7.67,-0.21)**	
	No (ref)	–	–	–	–	
**Patient has urine bag**	Yes	−10.66 (7.26)	(− 24.96, 3.64)*	NS	NS	
	No (ref)					
**Adjuvant therapy**	Chemotherapy	1.833(3.23)	(−4.53,8.19)			
	Radiotherapy	- 0.52(2.00)	(−4.46,3.42)			
	Combination	- 2.68 (1.59)	(−5.82,0.44)*	NS	NS	
	No Adjuvant Therapy (ref)	–	–			
**Social Support**	<=18 low social support	−10.67(2.42)	(−15.44, −5.90)*	− 5.66(2.01)	(−9.62,-1.71)**	
	> 18 high social support (ref)	–	–	–	–	
**Depression**	0–7(normal) (ref)	–	–	–	–	
	8–10(borderline abnormal)	- 7.32 (2.17)	(−11.60, −3.03)*	- 4.39 (2.03)	(−8.41,-0.39)**	
	11–21 (case abnormal)	- 21.80 (2.01)	(−25.76,-17.84)*	- 16.95 (2.04)	(−20.98,-12.93)**	
**Anxiety**	0–7(normal) (ref)	–	–	–	–	
	8–10(borderline case)	−7.77 (3.67)	(−15.01, −0.53)*	- 6.58(2.97)	(−12.43,-0.73)**	
	11–21(case abnormal)	−23.179 (3.96)	(− 30.983, −15.375)*	− 11.03 (3.47)	(− 17.88, −4.18)**	
Formal schooling * Role in the family	Formal schooling * Head/ decision makers of the family (ref)			–	–	
	No formal schooling * Not the head/ decision makers of the family	NA	NA	−0.32(3.08)	(−5.76,6.39)	
	No Formal schooling * Not head/ not decision makers of the family			−16.45(5.40)	(−27.08,-5.82)**	

R^2^ = 0.49

*Significant at *p* value < 0.025 by univariate linear regression

** Significant at *p* value < 0.05 by multivariable linear regression

NS (non-significant)

NA (not-applicable)

On univariate analysis (Table 4a) we observed that the resilience score was 3 units lower among participants with no formal education versus those with formal education. The resilience score of participants who were not head/not decision maker of the family was 5 units lower as compared to those who were the head/decision makers of the family. We also observed that the resilience score were 0.2 units lower among participants with household members more than 6 versus those with less than equal to 6. Smokeless tobacco users had 10 units higher resilience score as compared to ex-users. Moreover, resilience scores of participants with a family history of cancer were 4 units lower as compared to those with no F/H of cancer. Similarly, participants with F/H of depression had resilience score 6 units lower as compared to their counterparts. Participants with feeding tube and tracheotomy had resilience score 3 and 7 units lower as compared to those who did not respectively. Similarly, those with urine bag had resilience score 11 units lower as compared to those who did not. We also observed that the resilience score of participants with less social support were 11 units lower as compared to those who had high social support. Participants who were severely depressed had 21 units lower resilience scores as compared to those who did not have depression. Similarly, lower resilience scores were observed among those who were severely anxious as compared to those who were not anxious.

On Multivariable analysis (Table 4b) depression, anxiety, social support, family members in the household, smokeless tobacco users, tracheotomy and interaction between education and role in the family were significantly associated with resilience. After adjustment for the covariates we observed that the resilience scores of participants with more than 6 household family members was 2 (95% CI; − 4.31,-0.29) units lower as compared to those with ≤6 household family members. Moreover, the resilience score among current smokeless tobacco users was 10 (95% CI; 5.79, 14.45) units higher as compared to ex smokeless tobacco users. Resilience score among participants with tracheotomy was 4 (95% CI; − 7.67,-0.21) units lower as compared to those without it. In addition, resilience score among participants with low social support was 6 (95% CI; − 9.62,-1.71) units lower as compared to those with high social support. Moreover, resilience score among participants with severe depression was 17 (95% CI; − 20.98,-12.93) units lower as compared to non depressed, similarly, resilience score among those with borderline depression was 4 (95% CI; − 8.41,-0.39) units lower as compared to those who were not depressed. Resilience scores of participants with severe anxiety was 11 (95% CI; − 17.88,-4.18) units lower as compared to those with no anxiety, similarly, resilience score of those with mild anxiety was 7(95% CI; − 12.43, − 0.73) units lower as compared to those who were not anxious. There was a significant interaction between formal education and role in the family. Resilience score of participants who acquired no formal education and were not the head/ decision makers of the family was 16 (95% CI; − 27.08, − 5.82) units lower as compared to those who had formal education and were the head/ decision makers of the family.

## Discussion

This study aimed to assess the resilience of head and neck cancer survivors and its associated factors in Pakistani population. In our study population social support, number of family members in household, tracheotomy, depression, anxiety and smokeless tobacco were significantly associated with resilience.

In the present study we found an association between social support and high resilience. In Pakistan collectivistic culture prevails where family ties are greatly promoted. Social support provided by family members and healthcare professionals together help cancer patients in coping with the downside of their condition. It buffers against the negative impact of stress [31] instills hope and has positive effect on increase in lifespan [32, 33] which in turn promotes resilience [34]. Few studies report that during the ailment friends and family drift away [35] whilst others report that optimistic nature and adaptive coping strategies of patient fosters social support [36]. Pinar G et al. [31] and Brix et al. [37] found that there was negative association between need for psychosocial support and resilience as individuals with low resilience need psychosocial support in comparison to those having high resilience. Resilience plays protective role by minimizing the negative impact of distress [38, 39]. In our study participants with severe and borderline depression had lower resilience scores. Similarly participants with severe anxiety had lower resilience scores in comparison to those with mild anxiety or those who weren’t anxious. Brian et al. suggests that head and neck cancer patients should be routinely screened for depression and treated at the earliest to improve resilience [40].

Resilience was higher in cancer survivors having less than 6 household family members than those with at least 6 family members living with them. This finding is comparable to Narchal et al. [41] who also reported that small families comprising of 4 to 5 individuals have stronger family ties in comparison to those having large family size. The resilience of the family eases the burden on the principal caregiver and promotes the resilience of the cancer survivor [42]. In Pakistan the joint family system is more common where two or three generations reside under one roof [43]. With more family members living in the household the chances of emotional disclosure is higher than in nuclear family structures which might result in higher resilience [44] but at the same time large families residing in one household have multiple needs [45].

Resilience was lower in cancer survivors who had tracheotomy tube (to secure airway or difficulty with secretions) than those who did not. Tracheotomy adversely impacts the life of cancer survivors as their daily physical activities are restricted, speech is limited, physical appearance is altered and tracheostomy tube requires constant care. As dyspnea increases in head and neck cancer patients there is a dip in their resilience [46]. In contrast studies report that post treatment resilience improves significantly with every passing year as cancer survivors adapt to this new way of life [47–49]. In view of these findings it’s important that patients with tracheostomy are brought to a level where they can perform self-care [50].

Another finding of this study is that among cancer survivors the resilience score is higher among current smokeless tobacco users as compared to those who quit smokeless tobacco post treatment The findings of our study were consistent with those of an Indian study that found a significant positive correlation between smokeless tobacco use and QoL and a negative correlation with distress among head and neck cancer patients after treatment [51]. The plausible explanation of this could be that some cancer survivors manage anxiety of cancer recurrence by continuing tobacco consumption post cancer treatment as a coping strategy [52].

Formal education and decision maker of the family were seen as effect modifiers. The association of formal education and resilience varies by level of this third factor, role in the household decision-making process. Pakistan being a patriarchal society, where men hold greater authority in the household, women have limited decision making power but education has significant effect on decision-making power in the household irrespective of gender. Formal education results in a better job in turn more contribution to the household expenses thus an elevated role in the decision-making of the household resulting in higher resilience [52].

### Limitations and strengths

The strength of our study was that the data collectors were trained psychologist and provided on the spot counseling to patients identified as mildly anxious or depressed whereas those with symptomatic anxiety or depression were referred to seek advice from a psychiatrist. The setting of our study is Aga Khan University Hospital (AKUH), Karachi, the largest private tertiary care hospital in the largest metropolitan city of our country that caters to diverse ethnicities and socioeconomic groups thus a representative sample generalizing our findings.

There were some limitations in the present study, because of the cross sectional design temporality between resilience and mental health in head and neck cancer patients couldn’t be established. Longitudinal studies are needed to explore the association of resilience and mental health during the course of cancer treatment.

## Conclusion

In Pakistan, a South Asian LMIC, collectivist culture prevails, family ties are greatly promoted thus resilience and social support is highly prevalent in head and neck cancer patients resulting in lower prevalence of depression and anxiety. Our study highlights that higher resilience is prevalent among small families, less than six members, as the welfare of the individual is prioritized over multiple needs of the family. Formal Education and role in household/decision making power are effect modifiers in our study, demonstrating protective effect on mental health in head and neck cancer patients. High resilience scores were reported among current smokeless tobacco users as compared to quitters post treatment. Resilience-building interventions should be formulated to aidhead and neck cancer patients to cope with the disease and its sequelae.

## Supplementary Information

**Additional file 1.**

## Data Availability

The data that support the findings of this study are available from the corresponding author upon reasonable request.

## Abbreviations

LMIC: Lower and Middle Income Country
AKUH: Aga Khan University Hospital
CVD: Cardiovascular Disease
COPD: Chronic Obstructive Pulmonary Disease
RS-14: Resilience Scale 14
HADS: Hospital Anxiety and Depression Scale
ESSI: Enriched Social Support Instrument
CVI: Content Validation Index
IQR: Interquartile range
