# Supplementary material for: Resilience and its associated factors in head and neck cancer patients in Pakistan: an analytical cross-sectional study
Source: BMC Cancer. 2021 Aug 3;21:888. doi: 10.1186/s12885-021-08624-8 (PMC8330007; doi:10.1186/s12885-021-08624-8)
Supplement: Supplementary file 1 — Additional file 1. [file 12885_2021_8624_MOESM1_ESM.doc]

# *Resilience and Quality of Life of Head & Neck and Brain Cancer Survivors in Pakistan*

**Script: Now, through this form, I will be asking you questions on your socio-demographic factors, about your anxiety and depression, you’re coping behaviour, social support, quality of life and some explanatory questions. This is a self-administered tool but as all my participants might not be able to fill it by themselves so to maintain homogeneity; I will ask the following question and mark their response. The information obtained from you will be kept confidential and you may opt to not respond to any one or all the questions.**

***SOCIODEMOGRAPHY AND CLINICAL CHARACTERISTICS FORM***

**Section A: Socio-demography**

| **CODE** | **ITEMS** | **RESPONSE** | | | | | **Remarks (If Any Please Write Here)** |
| --- | --- | --- | --- | --- | --- | --- | --- |
| A.1 | How old are you? (In years) |  | | | | |  |
| A.2 | Gender | 1. Male | | | |  |  |
|  |  | 1. Female | | | |  |  |
| A.3 | Have you ever had formal schooling? | 1. Yes | | | |  |  |
|  |  | 1. No (skip to 4) | | | |  |  |
| A.3a | How many years of formal schooling? (In years) |  | | | | |  |
| A.4 | Have you had any type of informal education | 1. Yes | | | |  |  |
|  |  | 1. No (skip to 5) | | | |  |  |
| A.4a | Which type? (Mark all that apply) | 1. Madrassa | | | | |  |
|  |  |  |  |  |  |  |  |
|  |  | 1. Adult literacy | | | | |  |
|  |  |  |  |  |  |  |  |
|  |  | 1. Home schooling (male/female teacher) | | | | |  |
|  |  |  |  |  |  |  |  |
|  |  | 1. Self learnt (reading) | | | | |  |
|  |  |  |  |  |  |  |  |
|  |  | 1. Self learnt (reading and writing) | | | | |  |
|  |  |  |  |  |  |  |  |
|  |  | 1. Learnt Quran | | | | |  |
|  |  |  |  |  |  |  |  |
|  |  | 1. Other : | | | | |  |
|  |  | (SPECIFY):_____________ | | | | |  |
| A.5 | What is your mother tongue? | 1. Sindhi | |  | | |  |
|  |  | 1. Urdu | |  | | |  |
|  |  | 1. Punjabi | |  | | |  |
|  |  | 1. Marwari | |  | | |  |
|  |  | 1. Saraiki | |  | | |  |
|  |  | 1. Pushto | |  | | |  |
|  |  | 1. Balochi | |  | | |  |
|  |  | 00. Other | |  | | |  |
|  |  | (SPECIFY):_____________ | | | | |  |
| A.6 | Marital status | 1. Single  2. Married |  | | | |  |
|  |  |  |  |  |  |  |  |
|  |  | 3. Divorced |  | | | |  |
|  |  | 4.Widowed/Widower |  | | | |  |
| A.7 | What is your family type | 1. Nuclear |  | | | |  |
|  |  | 1. Extended |  | | | |  |
| A.8 | What is your role in family | 1. Head |  | | | |  |
|  |  | 1. Not Head but take part in decision | | | | |  |
|  |  |  |  |  |  |  |  |
|  |  | 1. Does not take decision, only follower | | | | |  |
|  |  |  |  |  |  |  |  |
| A.9 | How many people live in your household |  | | | | |  |
| A.10 | Are you currently working? | 1. Yes | | |  | |  |
|  |  | 1. No (skip to 11) | | |  | |  |
| A.10a | If yes, please describe your occupation? |  | | | | |  |
| A.11 | Is your husband/wife employed? | 1. Yes | | |  | |  |
|  |  | 1. No (skip to 12) | | |  | |  |
| A.11a | If yes, describe the type of work that he/she does |  | | | | |  |
| A.12 | Total household monthly income (in PKR) |  | | | | |  |

**Section B: Co-Morbid Condition**

| **CODE** | **ITEMS** | **RESPONSE** | | | | | **REMARKS (if any please write here)** |
| --- | --- | --- | --- | --- | --- | --- | --- |
| B.1 | Do you have Hypertension? | 1. Yes | | |  | |  |
|  |  | 1. No (skip to B 2) | | |  | |  |
| B.1a | Since how many years? |  | | | | |  |
| B.2 | Do you have Diabetes? | 1. Yes | | |  | |  |
|  |  | 1. No (skip to B3) | | |  | |  |
| B.2a | Since how many years? |  | | | | |  |
| B.3 | Do you have Cardio vascular Disease? | 1. Yes | | |  | |  |
|  |  | 1. No (skip to B 4) | | |  | |  |
| B.3a | Since how many years? |  | | | | |  |
| B.4 | Do you have Kidney Problem? | 1. Yes | |  | | |  |
|  |  | 1. No (skip to 5) | |  | | |  |
| B.4a | Since how many years? |  | | | | |  |
| B.5 | Do you have Liver Problem? | 1. Yes |  | | | |  |
|  |  | 1. No (skip to B6) |  | | | |  |
| B.5a | Since how many years? |  | | | | |  |
| B.6 | Have you undergone any surgery other than head and neck surgery? | 1. Yes | | | |  |  |
|  |  | 1. No (skip to Section C) | | | |  |  |
| B.6a | If yes, please specify |  | | | | |  |

**Section C: Addiction History**

| **CODE** | **ITEMS** | **RESPONSE** | | | **REMARKS (if any please write here)** |
| --- | --- | --- | --- | --- | --- |
| C.1 | Do you smoke cigarettes? | 1. Yes | |  |  |
|  |  | 1. No (skip to Q C2) | |  |  |
|  |  | 1. Ex-Smoker | |  |  |
| C.1a | Since how many years? |  | | |  |
| C.1b | How many cigarettes do you smoke in a day? |  | | |  |
| C.2 | Do you use smokeless tobacco (Chalia, Paan etc)? | 1. Yes |  | |  |
|  |  | 1. No   (skip to Q C3) |  | |  |
|  |  | 1. Ex-Smoker |  | |  |
| C.2a | Since how many years? |  | | |  |
| C.2b | Which type of smokeless tobacco do you use? |  | | |  |
| C.2c | How many do you use in a day? |  | | |  |
| C.3 | Do you drink Alcohol? | 1. Yes |  | |  |
|  |  | 1. No (skip to Section D) |  | |  |
|  |  | 1. Ex alcohol user |  | |  |
| C.3a | Since how many years? |  | | |  |
| C.3b | How many glasses do you drink in a day? |  | | |  |

**Section D: Family History**

*(Note: Family refers to close relative including parents, siblings, grandparents and maternal & paternal aunty and uncle)*

| **CODE** | **ITEMS** | **RESPONSE** | | | | **REMARKS (if any please write here)** |
| --- | --- | --- | --- | --- | --- | --- |
| D.1 | Does anyone in your family have head and neck cancer? | 1. Yes | |  | |  |
|  |  | 1. No (skip to D 2) | |  | |  |
| D.1a | If yes, Who? |  | | | |  |
| D.2 | Does anyone in your family have any other cancer than head and neck cancer ? | 1. Yes |  | | |  |
|  |  | 1. No (skip to D3) |  | | |  |
| D.2a | If yes, which type of cancer |  | | | |  |
| D.2b | If yes, who has it? |  | | | |  |
| D.3 | Does anyone in your family have depression? | 1. Yes | | |  |  |
|  |  | 1. No (skip to Section E) | | |  |  |
| D.3a | If yes, who has it? |  | | | |  |

**Section E: Major Life Events**

| **CODE** | **ITEMS** | **RESPONSE** | | | | **REMARKS (if any please write here)** |
| --- | --- | --- | --- | --- | --- | --- |
| E.1 | Any deaths has occurred in family in the last 6 months | 1. Yes | | |  |  |
|  |  | 1. No (skip to E 2) | | |  |  |
| E.1a | Who died | 1. Child |  | | |  |
|  |  | 1. Spouse |  | | |  |
|  |  | 1. Parents |  | | |  |
|  |  | 1. In-laws |  | | |  |
|  |  | 00.Other |  | | |  |
|  |  | (SPECIFY):_____________ | | | |  |
| E.1b | How did the death occur: Specify |  | | | |  |
| E.2 | Any kidnapping of Family member in the last 6 months | 1. Yes | |  | |  |
|  |  | 1. No | |  | |  |
| E.3 | Any theft occurred in the last 6 months | 1. Yes | |  | |  |
|  |  | 1. No | |  | |  |

**Section F: Clinical Characteristics**

| **CODE** | **ITEMS** | **RESPONSE** | | **REMARKS (if any please write here)** |
| --- | --- | --- | --- | --- |
| F.1 | Site of tumour | 1. Head and neck cancer | |  |
| F.2 | Types for Head and neck cancer | 1. Oral cancer 2. Oropharyngeal 3. Nose and paranasal sinus 4. Nasopharyngeal 5. Hypopharyngeal 6. Laryngeal 7. Salivary gland 8. Thyroid cancers 9. Buccal 10. Tongue 11. Others: Specify | |  |
| F.3 | Date of diagnosis of tumor/ cancer |  | |  |
| F.4 | Surgical intervention | 1. Biopsy 2. Total Resection 3. Sub-Total Resection 4. No surgical intervention | |  |
| F.5 | Treatment of cancer/tumor | 1. On-going 2. Complete | |  |
| F.6 | Do you have tube feed? | 1. Yes |  |  |
|  |  | 1. No |  |  |
| F.7 | Does the patient have tracheostomy? | 1. Yes |  |  |
|  |  | 1. No |  |  |
| F.8 | Does the patient have urine bag? | 1. Yes |  |  |
|  |  | 1. No |  |  |
| F.9 | Adjuvant therapy | 1. Chemotherapy 2. Radiotherapy 3. Combination 4. No Adjuvant Therapy | |  |
